# Discovering NDM-1 inhibitors using molecular substructure embeddings representations

**DOI:** 10.1515/jib-2022-0050

**Published:** 2023-07-28

**Authors:** Thomas Papastergiou, Jérôme Azé, Sandra Bringay, Maxime Louet, Pascal Poncelet, Miyanou Rosales-Hurtado, Yen Vo-Hoang, Patricia Licznar-Fajardo, Jean-Denis Docquier, Laurent Gavara

**Affiliations:** LIRMM, University of Montpellier, CNRS, 34095 Montpellier, France; AMIS, Paul Valery University, 34199 Montpellier, France; IBMM, CNRS, University of Montpellier, ENSCM, 34293 Montpellier, France; HSM, University of Montpellier, CNRS, IRD, CHU, Montpellier, France; Department of Medical Biotechnologies, University of Siena, I-53100 Siena, Italy

**Keywords:** drug discovery, machine learning, multiple instance learning, NDM-1 inhibitors

## Abstract

NDM-1 (New-Delhi-Metallo-β-lactamase-1) is an enzyme developed by bacteria that is implicated in bacteria resistance to almost all known antibiotics. In this study, we deliver a new, curated NDM-1 bioactivities database, along with a set of unifying rules for managing different activity properties and inconsistencies. We define the activity classification problem in terms of Multiple Instance Learning, employing embeddings corresponding to molecular substructures and present an ensemble ranking and classification framework, relaying on a k-fold Cross Validation method employing a per fold hyper-parameter optimization procedure, showing promising generalization ability. The MIL paradigm displayed an improvement up to 45.7 %, in terms of Balanced Accuracy, in comparison to the classical Machine Learning paradigm. Moreover, we investigate different compact molecular representations, based on atomic or bi-atomic substructures. Finally, we scanned the Drugbank for strongly active compounds and we present the top-15 ranked compounds.

## 1 Introduction

**Figure 1:**
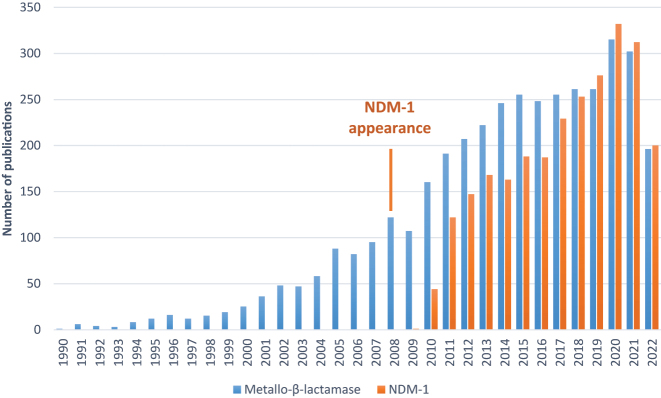
Number of publications by year with “Metallo-β-lactamase” (blue) and “NDM-1” (orange) terms indexed by PubMed©.

Due to human way of life and overuse of antibiotics, the bacterial resistance is growing up every day and is spreading across the world [1]. The main therapeutic class involved against bacterial infections is based on the penicillin core: the β-lactams. This class shares a common structural moiety: a four-membered β-lactam ring, essential for the biological activity [2]. The main mode of bacterial resistance in case of Gram-negative pathogens is mediated by the expression of enzymes able to hydrolyze this crucial ring: the β-lactamases [3]. They are classified into 4 molecular classes (A, B, C and D) but can be divided into two main categories based on their mechanism of action. The first group of enzymes described were the serine β-lactamase enzymes (SBLs, classes A, C and D) and several inhibitors such as β-lactam, DBO or more recently boron-based inhibitors have been developed and are currently used in combination with β-lactam agents [4]. Three decades ago, a new class of β-lactamases, named Metallo-β-Lactamases or MBLs (class B) has emerged [5]. They are characterized by the presence of one or two zinc atoms into the active site, acting as Lewis acids, that increase the electrophilicity of the azetidinone ring, while permitting the deprotonation of the nucleophile, a water molecule. At the beginning, these enzymes were considered as biochemical curiosities but now, they are recognized as the most worrying threat to bacterial disease treatments. Indeed, MBLs are able to inactivate a broad-spectrum of β-lactams including carbapanems, already restricted to severe infections in hospitals [6]. Because of a different catalytic mechanism compared to SBLs, the SBL inhibitors are inefficient on MBLs and there is no inhibitor available yet on the market. Among numerous characterized MBLs, NDM-1 (New-Delhi-Metallo-β-lactamase-1), emerged in India in 2008 [7] and spread quickly worldwide to be present everywhere now, has become the most common MBL subtype in numerous countries and the most studied too (Figure 1). Indeed, NDM-1 is able to hydrolyze almost all families of β-lactam agents (except monobactam) including last resort antibiotics, the carbapenems [8]. Many variants arose around the world, but NDM-1 subtype remains the most prevalent. NDM-1-producing pathogens (also called super-bugs) represent one of biggest threat on human health [9] and it’s crucial to address this major trouble especially by the development of specific and potent inhibitors.

Even if no NDM-1 inhibitors are currently marketed, numerous studies in the literature describe effective compounds [10]. It’s possible to classify them into 3 different modes of action [11]. The first type of inhibitors are zinc-chelating agents targeting the fundamental zinc atom requirement of the metallo-enzyme for its catalytic activity. The first described inhibitor was EDTA, a strong and non-specific chelating agent acting through a metal stripping mechanism [12]. The most promising agent of this category is Aspergillomarasmine A (AMA), a natural product highly active *in vitro* and *in vivo* against NDM-1 producers [13]. The second category of compounds falls into the major category of competitive inhibitors, targeting the active site. These molecules are often characterized by the presence of chemical functional groups able to establish strong ionic bonds with zinc atoms, into the catalytic pocket, such as thiol, carboxylic acid, or any other acidic hydrogen atoms. One of the best examples is the thiazole ANT2681, a selective NDM-1 inhibitor [14], that successfully completed preclinical stage. The last possibility, and the less common, is allosteric inhibitors defined by compounds able to bound to a specific surface of the biological target inducing conformational modifications that disturb the enzymatical activity [15].

Despite all these efforts, the development of a marketed NDM-1 inhibitor drug remains an unmet therapeutic need and must be addressed. Nevertheless, the process of drug discovery is a highly time-consuming (between 10 and 14 years) and extremely expensive (1 billion USD magnitude) procedure, characterized by high-level attrition rates, to reach marketing approval [16]. The first step in medicinal chemistry is to identify new candidate compounds that will be subsequently synthesized and tested *in vitro* against a specified target. For this purpose *in silico* methods (i.e. Virtual Screening [VS]), have be widely used for the identification of prominent compounds speeding-up thus, drug discovery. Virtual Screening techniques can be identified in three major categories: (i) structure-based approaches where the 3D structure of the target should be known, and which involve mainly docking procedures; (ii) ligand-based VS where knowledge of the active ligand is required, and which involve, mainly, Quantitative Structure-Activity Relationships (QSAR) modeling or substructure/similarity searching; and (iii) hybrid approaches that combine the two former VS approaches [17]. In the last years, as the availability of open-access ligand databases (e.g. ZINC 15 [18], ChEMBL [19] etc.) has been significantly increased, new ligand-based approaches based on Machine Learning (ML) and Deep Learning (DP) have been proposed. Efficient ML models for hit identification (i.e. identification of potent small compounds for starting a medicinal chemistry pipeline), drug repurposing, activity scoring [20] or activity prediction [21] showed significant performance. For using efficiently a data-driven approach for such tasks (e.g. activity prediction), there is a need for specialized and annotated data (e.g. ligand-activity data) that refer to a specific target, since models built on general data (e.g. antibacterial, anti-cancer, anti-inflammatory activity data) will perform poor when it comes on specific tasks, like the discovery of potent NDM-1 inhibitors. A successful story in this field is the Halicin identification by a deep learning approach as new antibiotic agent [22]. Based on a library of 2335 active or inactive compounds, mixing several kinds of modes of action, the study identified this potent antibacterial molecule with completely original biological mechanism, thank to drug repurposing strategy. Currently, the Halicin development is ongoing at a preclinical stage.

In this work, Machine Learning models are employed to introduce a framework for discovering compounds that have potentially strong activity against NDM-1. To this end, (i) we introduce a new database of 868 compounds collected from the recent literature, bearing experimental NDM-1 activity data, as well as considering only relevant compounds from the NDMI database [23]; (ii) we introduce a comprehensive procedure for annotating compounds in three classes (no-activity, weak activity, strong activity against NDM-1) based on different activity experiment outcomes, which can handle and cure inconsistencies caused by contradictory reported properties; (iii) we define the activity classification problem in the frame of Multiple Instance Learning employing substructure-based molecular embeddings; and (iv) we introduce and evaluate a homogeneous ensemble classification and per class ranking framework for the 3-class NDM-1 activity classification problem.

We resume the contributions of our work:

1. We introduce a new sanitized NDM-1 activities database, labeled in a consistent manner, using a comprehensive procedure based on experimental activity outcomes.
2. The definition of the activity classification problem as a 3-class Multiple Instance Learning problem, where molecules are represented as a collection of Mol2vec embeddings corresponding to molecular substructures of different radii. MIL classification shows significant better classification performance then the classical Mol2vec embeddings that correspond to a whole molecule.
3. We introduce a classification and ranking framework that consists of an ensemble of homogeneous classifiers, which achieves comparable, to the initial classifier, results when assessed on an independent test set, highlighting thus the generalization ability of the ensemble Multiple Instance Learning model. Furthermore, the ranking evaluation of the aforementioned MIL framework demonstrates promising results, especially for the inactive and strongly active classes, achieving 100% top-3 and top-5 accuracy for the strongly active class.
4. We performed a series of experiments on different kinds of MIL molecular representations, using substructure embeddings involving different radii of substructures, demonstrating that the representation of molecules using substructures of radius 1 (atoms and their neighbors) can be beneficial to the overall classification and ranking performance of the MIL models.
5. Finally, using the proposed classification and ranking framework we preformed Virtual Screening on the DrugBank [24], where we classified and rankinged 11,290 drugs. The VS procedure ranked the strongly active compounds (according to the classification), identifying 6 experimental, 1 investigational and 3 approved drugs, among the top-10 ranking results of the strongly active class.

## 2 Related works

Machine Learning has, in the last years, broadly employed in the field of drug design. Various applications has been proposed, including protein-drug interaction predictions, drug potency discovery, biomarkers safety assessment, protein folding prediction, protein-to-protein interactions prediction, drug repurposing, hit identification etc. [20]. Machine Learning models have been also used for molecular properties predictions including bioactivities, bio-distribution and physical molecular properties [25]. In [26] Lee et al. proposed a random matrix theory inspired methodology, coupled with high-quality negative data, for identifying compounds active against the human muscarinic acetylcholine receptor M1, a receptor that relates to Alzheimer’s disease and schizophrenia. In [27] Mayr et al. conducted a large-scale comparison of Machine Learning models on a variety of activity classification problems extracted from ChEMBL. In their work, they compare Depp Neural Networks (i.e. Feed-forward Neural Networks – FNN), Convolutional Neural Networks (Graph Convolution [GC] and Weave from the DeepChem package [28]) and Long-Short-Term Memory (LSTM) networks operating on molecular string representations (SMILES). Furthermore, they included in their investigation classical Machine Learning models like Support Vector Machines (SVM), k-Nearest Neighbors (k-NN), Naïve Bayes classification and Similarity Ensemble Approach (SEA). In [23] Shi et al. composed a NDM-1 activities dataset, that included strong and weak active compounds of established activity against NDM-1, as well as a collection of “hypothetical” non-active compounds, selected based on physicochemical features. In their study, they compared the efficiency of Machine and Depp Learning models that were based on molecular descriptors derived from MOE2018,1
https://www.chemcomp.com/Products.htm. in the task of the 3-class activity classification problem.

A category of Machine Learning models that falls in the domain of weakly supervised learning, is Multiple Instance Learning (MIL). In Multiple Instance learning, the objects (i.e. samples to be classified) are not represented by a single vector (as in classical Machine Learning) but by a collection of multiple vectors, each one representing a different aspect of the object. In this frame, the objects are called bags and the elements of the bags are called instances. Multiple Instance Learning is a paradigm of weakly supervised learning, since labels are provided for the bags and none information is provided on the annotation of the individual instances. In this framework, a bag can consist of instances having different latent annotations of instances, e.g. an active molecule (i.e. bag) can contain instances that are both active and inactive.

Dietterich et al. [29], introduced in 1997 in their seminal paper, Multiple Instance Learning for the first time, for dealing with the problem of the prediction of the binding of a compound to a musk receptor. Each molecule can take different conformations and some of them can bind to the musk receptor while others not. For this reason, each molecule (i.e. bag) is represented by a collection of different vectors corresponding to different conformations. Thus, the standard MIL assumption was defined stating that a negative (i.e. inactive) bag can only contain inactive instances while a positive (i.e. active) bag must contain at least one positive instance. Multiple Instance Learning has been successfully applied in different areas comprising classification of medical images, frailty prediction by monitoring physiological signals [30], classification of natural images [31, 32] and drug discovery [33].

More specifically, Bergeron et al. [34] proposed a bundle algorithm for optimizing the nonconvex Multiple Instance Learning objective function, tackling under others the problem of bioavailability of drugs. El-Manzalawy et al. [35] formulate the problem of predicting qualitatively and quantitatively flexible length Major Histocompatibility Complex Class II (MHC-II) molecules as MIL and MIL regression problems, an important task for the development of novel vaccines. Bandyopadhyay et al. in ref. [36] propose MBSTAR for the prediction of true or functional microRNA binding sites by handling the absence of information on physical binding sites of the targeted mRNAs. Finally, Eksi et al. [37] developed an Multiple Instance Learning framework for predicting gene functions.

Numerical molecular representations play a decisive role in the process of building Machine Learning models for the different cheminformatics tasks, and affect significantly the models’ performance. Different molecular representations have been proposed, which include Fingerprints (Extended-Connectivity Fingerprints, a.k.a. Morgan Fingerprints), molecular graphs or other computer learnt representations [38]. Such a representation, inspired by the Natural Language Processing word2vec model, is the Mol2vec representation [39]. Mol2vec considers molecular substructures, based on Morgan Fingerprints, as words and molecules as sentences of substructures obtaining thus molecular embeddings by training a word2vec model.

## 3 Architecture/implementation/workflow

**Figure 2:**
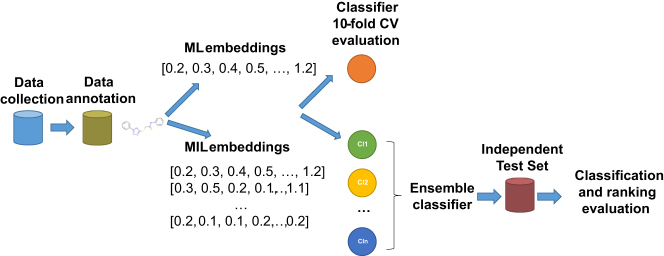
The workflow of the study.

The workflow of our work is summarized as follows. In the first phase, data collection takes place, where NDM-1 activity data of compounds are collected and sanitized. In a second phase, data are annotated using a unifying procedure that can handle contradictions and inconsistencies. Subsequently, the embeddings for the ML and MIL cases are calculated and the classifiers are trained and evaluated. Finally, the ensemble classifier is built and its classification and ranking performance is evaluated on a independent held-out test set. Figure 2 resumes the whole workflow of this study.

### 3.1 Dataset collection

To achieve the global objective, (i.e. *in-silico* identification of NDM-1 inhibitors), it is necessary to generate the largest database of compounds based on all published literature outputs. Some databases of NDM-1 inhibitors have been already established through publications and/or websites [23, 40]. Unfortunately, all of the existing databases were largely incomplete and in some cases they incorporated structural or biological mistakes. Good quality data are imperative for building a good performing predictive model. To this end and for avoiding any bias or flaws, a sequential approach was adopted,which included a research by keywords on devoted websites (PubMed© and SciFinder©), and the main scientific editors (ACS, Elsevier, RSC and Willey) as well as the study of the most recent reviews on the topic [10, 41, 42]. At this point, a methodology was established to prevent the introduction of duplicate compounds. There are several ways to convert chemical structures into computer usable files including graph or linear representations [43]. We selected the SMILES (Simplified Molecular Input Line Entry Specification) representation , where each molecule is represented by a small string of characters [44]. Each SMILES string corresponds to only one chemical structure, but each molecule possesses generally several different SMILES. Nevertheless, it’s possible to convert SMILES in the canonical SMILES format, where each molecule corresponds to an unique canonical SMILES representation, to detect duplicates [45]. Another advantage of the SMILES representation is that it is not a hashing function and each part of the string represents a part of the molecule. It is even possible to use it for substructure research as well as for the correct identification of what is important or not in a specific interaction with a biological target.

All structures, along with their corresponding biological activities data, were collected manually and converted in canonical SMILES strings with RDKit.2
https://www.rdkit.org/. In case of duplicate compounds with different biological values, only the best activity for the compound was retained, according to the ranking procedure described below. Thus, a database of 868 unique compounds were generated from 82 corresponding publications identified by their unique doi number. As introduced previously, NDM-1 inhibitors can display several possible modes of action and for the majority of them, the exact mechanism is not provided or demonstrated. Indeed, the rigorous determination of the mode of action needs a lot of efforts involving specific experiments and can lead to inconclusive or contradictory results. Fortunately, as Halicin identification showed, the knowledge of the action mechanism is unnecessary for reaching our goal, and no mechanism distinction was introduced in the database.

### 3.2 Labeling the database

The next step consists in the assessment of the biological activities data for each previously identified molecule. To evaluate biological properties of compounds, several assays are available leading to different values. To meet the desired quality of our final database, it is necessary to classify and rank the different available biological properties. At the beginning, the properties are divided between enzymatic and bacterial models, with Minimal Inhibitory Concentration (MIC) being the only bacterial considered property. In the case of bacterial experiments in microbiology, the biological effect is generally determined by the Minimum Inhibitory Concentration (MIC). In the specific scenario of NDM-1 inhibitors, MIC value is an indirect measurement of the adjuvant effect to protect the real active agent: the β-lactams. Moreover, MIC values are sensitive to experiment protocol and more vulnerable due to the complexity of the model and the number of variables. For these reasons, we ranked the bacterial properties (MIC) in the last place, meaning that we will use them only if no other enzymatic property is available. The inhibitor constant (*K*_i_) is probably the most enzymatically accurate value, because it’s not dependent on the experimental conditions, but it can be determined only on competitive inhibitors, and it is experimentally more difficult to obtain. Very often, half maximal inhibitory concentration (IC_50_) is preferred (or its counterpart pIC_50_), because it’s easier to determine and can be used on every kind of inhibitors [46]. Unfortunately, the IC_50_ value depends on several experimental conditions such as the nature of the reporter’s substrate and its concentration. That means that the same compound can have quite different IC_50_ values according to the experimental procedures and that is why when we have both values, only *K*_*i*_ will be retained. Finally, as concerns the mono-concentration inhibition value (%100 µM) it can be set only at one defined concentration for an inhibitor. These assays exhibit the same limitations as for the IC_50_ determination, but with a bigger uncertainty. The mono-concentration inhibition value it is generally used only as screening method to determine which compound needs to be further biologically characterized. Thus, in our case, it will be only used if no other property is available to describe the biological activity of an inhibitor.

Since the labeling of the compounds will be based on the values of experimentally obtained properties and since in the literature several properties are possibly observed for a single compound, we need to come up with a procedure for automated selection of the best property for each compound (if several observed properties exist). To this end, we establish a decreasing importance ranking of the biological activity properties, which is summarized in Table 1. As explained above, enzymatic properties are ranked above properties based on bacterial assays and furthermore the enzymatic properties are ranked according to their accuracy and their independence with respect to the experimental conditions (the more accurate and independent properties, with respect to the experimental conditions, are ranked higher). For each compound only the value of the highest ranked property is considered, if several properties are observed. Furthermore, in order to deal with inconsistencies (e.g. the same compound having different observed values for the same property in different literature publications, due to various experimental conditions) we only consider the value that corresponds to the highest observed activity against NDM-1, since there is at least one evidence (i.e. one experiment) of this high activity. To summarize the unifying methodology: for each compound, only the biological activity with the highest ranking will be retained and in the case of 2 different values of a property of the same rank, the most active value will be preserved. We have to note here, that the observed inconsistences (i.e. different values reported for the same property of the same compound that could result to different labelling), which were resolved using the aforementioned procedure, comprise only a small part of the database: 25 compounds (i.e. 2.88 % of the database).

**Table 1:** Labeling cut-off scores of activity properties.

Rank	Activity property	Not active	Weakly active	Strongly active
1	*K*_i_ (µM)	>10	(0.5, 10]	≤0.5
2	IC_50_ (µM)	>20	(1, 20]	≤1
3	pIC_50_	<4.7	[4.7, 6)	≥6
4	%100 µM	<60 %	≥60 %	–
5	*K*_d_ (µM)	>10	(0.5, 10]	≤0.5
6	MIC (µg/mL)	>8	(0.5, 8]	≤0.5

For our approach, cut-off values have to be established, to group the inhibitors into 3 categories: Strongly Active (SA), Weakly Active (WA) and Not Active (NA). The definition of good or bad inhibition values depends on several parameters in the literature, such as the state-of-the-art in the corresponding field, the biological target or the experimental model of evaluation, largely influenced by personal point of view at every stage. To avoid arbitrary limits, the different thresholds must be defined for the 3 categories. Based on this objective, *K*_*i*_ cut-off values were set to 0.5 and 10 µM to define the limits between SA-WA and WA-NA respectively. IC_50_ and *K*_i_ values are related by the Cheng-Prusoff relationship ( $Ki=\frac{\text{IC}50}{1+\frac{\left[S\right]}{Km}}$ , where [*S*] stands for Substrate concentration and *K*_*m*_ for the Michaelis constant) and the IC_50_ thresholds result by setting the minimal value of 1 for the [*S*]/*K*_*m*_ ratio (generally, for experimental considerations, [*S*] is set at 3–4 times *K*_*m*_ value) [47]. Due to the relationship pIC_50_ = −log10 (IC_50_), the corresponding limits for pIC_50_ values can be easily calculated. Finally, the mono-concentration inhibition value (%100 µM) threshold is arbitrary set to 60 % and it is apllied only to a small part of the data (1.84 %) for determining the annotation. The cut-off values for each property and each class are summarized in Table 1.

### 3.3 Analysis of the database

**Figure 3:**
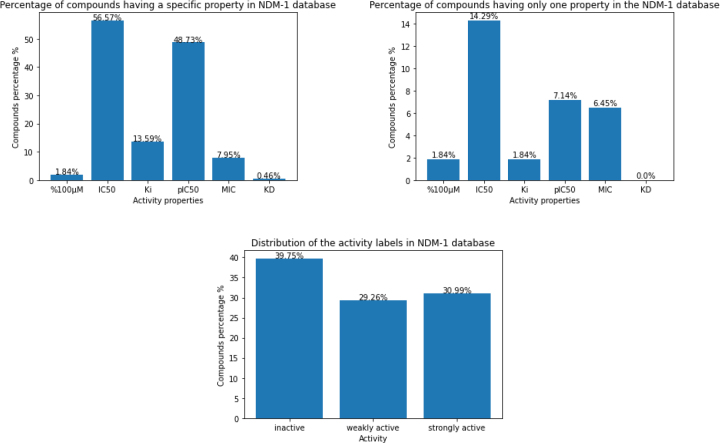
Percentage of compounds having a specific property (some compounds may have more than one properties) (upper left corner), percentage of compounds having only one property (upper right corner), distribution of the annotations of the compounds according to the procedure in Section 3.2 (lower row).

The compiled database consists of 868 compounds of known biological activities against NDM-1. After the annotation procedure, described in detail in Section 3.2, we obtained 345 inactive, 254 weakly active and 269 strongly active compounds. The percentage of the compounds belonging to different classes, as seen in Figure 3, makes the dataset relatively balanced. Furthermore, Figure 3 reports the percentage of the compounds having different properties (some compounds have more than one available properties), as well as the percentage over the total number of compounds that have only one available property. It can be observed that the majority of the compounds dispose enzymatic inhibition properties, compared to the compounds characterised by bacteria growth inhibition properties (MIC). Furthermore, for 31.56 % of the compounds only one property is available , on which we have to rely and, as a seen in Figure 3, for only 6.45 % of the compounds, we need to rely on MIC. Finally, there is no compound where we have to rely on *K*_d_ for annotating their inhibition capacity.

**Figure 4:**
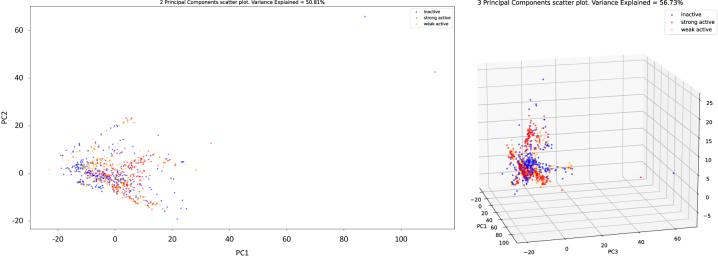
2D scatter plot of the 2 first principal components per class (left). 3D scatter plot of the 3 first principal components per class.

Figure 4, presents the 2D and 3D projections per class of the compounds, depicting the first two and three principal components, as obtained by the Principal Components Analysis of the compounds represented as Mol2vec vectors (see Section 3.4.1). The variance of the dataset explained is 50.81 % and 56.73 % for the 2D and 3D projections respectively.

### 3.4 Calculation of substructure embeddings

#### 3.4.1 Embeddings for machine learning algorithms

For representing each compound by a vector, we employed the Mol2vec embeddings [39]. For each molecule, identifiers corresponding to radii 0 and 1 are calculated using the Extended-Connectivity Fingerprints (a.k.a. Morgan) algorithm [48]. Each Morgan identifier represents a molecular substructure, with identifiers of radius 0, corresponding to individual atoms, along with their corresponding bonds; and identifiers of radius 1 corresponding to atoms, along with their neighbors. The identifiers are considered as words and a molecule, consisting of an ordered sequence of identifiers, is considered as a sentence. Rare identifiers are defined as identifiers occurring less than 3 times in the dataset, and marked by a special identifier, called Unknown (‘UKN’) identifier. Afterwards, a word2vec model is trained, using the skip-gram setting, for obtaining the Mol2vec embeddings for each identifier. For obtaining, finally, an embedding for each compound, the embeddings of the individual identifiers that constitute a molecule are added-up.

In our study, we employed a pre-trained Mol2vec model, producing 300 dimensional vectors. The model was trained using a window size of 10, Morgan identifiers of radii 0 and 1 and a database of 19.9 M molecules of the ZINC and ChEMBL databases.

#### 3.4.2 Embeddings for multiple instance learning algorithms

In the Multiple Instance Learning setting, each subject (i.e. a bag) must be represented as an unordered set of vectors (i.e. instances). In our study, we aim to represent each compound as a collection of its substructures. To this aim, we exploit the embeddings outputted by the word2vec model that correspond to individual substructures (Morgan identifiers) of each molecule. Thus, in the MIL setting, a molecule is represented by the collection of the embedding vectors corresponding to individual substructures. The bio-activity labels are provided for each molecule (i.e. bag) but the activities for each particular substructure (i.e. instance) are unknown. In this way, for a molecule to be active (i.e. to be able to inhibit NDM-1), at least one of its substructures must be involved in the binding affinity.

The Mol2vec model, as explained in Section 3.4.1, produces embeddings corresponding to molecular substructures and is able to represent a molecule using all available substructures up to a given radius *r*. As each molecule is considered as a sequence of identifiers, duplicate identifiers can be present in a molecule. In the MIL setting (i.e. bag representation of each molecule) such instances (i.e. vectors corresponding to the same substructure) do not contribute to the classification procedure, and thus are removed. We introduce two types of compounds’ representations in the MIL setting, an integral, comprising all available information on the molecule, and a more comprehensive representation. In the first case, we represent each compound by the unique embedding vectors, corresponding to all its radii 0, 1, 2, …, *r* substructures. Thus, a molecule is represented multiple times using substructures of different size. In the second case, the molecular representation is based only on the unique substructure embeddings that correspond to a specific radius *r*. Thus, a compound is represented only once, using substructures of a fixed length. In Section 4, we will evaluate the different representation methods to examine experimentally, which of these is contributing to a better classification performance.

The Multiple Instance Learning representation, unlike the Mol2vec representation, where a unique vector is produced by the addition of all the substructure vectors, includes explicitly all substructure embeddings. In Section 4, we will show experimentally, that the MIL representation has a positive effect in the classification performance in comparison to the classical ML compound representation. In fact, using the classical Multiple Instance Learning assumption for a non-active compound, we must expect that no substructures should contribute to the binding affinity (i.e. be non-active). In the case of weakly and strongly active compounds, we should expect that a fraction of its substructures should be active.

### 3.5 Classification and ranking

As a Virtual Screening procedure using a classifier, (e.g. for NDM-1 strong inhibitors identification, in a large compounds’ database), will attribute to all the compounds an activity class, a ranking procedure is necessary, for ranking per class the classification outputs in terms of the confidence of the classifier. For this purpose, we propose in this section a homogeneous ensemble classification and ranking scheme that is able to classify and in the same time rank the classification results per class. We define formally such a framework and specify the choice of the individual classifiers.

Let $f_{i}^{h_{i}}:R^{m}\to \left\{cl\text{\_}1,\ldots ,cl\text{\_}n\right\}$ , $d_{i}^{h_{i}}:R^{m}\to R$ , *i* = 1, …, *k*, being *k* distinct classifiers and the corresponding decision functions respectively, with *n* being the number of classes and *m* the number of features. We can choose these classifiers by performing a *k*-fold Cross Validation (CV) procedure using a hyper-parameter optimization procedure per fold. Let $h_{i}\in R^{l}$ be the hyper-parameters for each individual fold. Thus, we will obtain *k* classifiers that are trained in different training sets, having different hyper-parameters, which form a set of homogeneous classifiers. A voting procedure $g\left(f_{0}^{h_{0}},\ldots ,f_{k}^{h_{k}}\right)$ = *c* can provide the final output of the ensemble classifier. For calculating the confidence rank (i.e. highly ranked outputs are considered to have more confident classification) we calculate the mean of the decision values of the classifiers that contributed, according to the voting procedure, to the classification of an object. Thus the rank of a sample, inside the predicted class, can be calculated by $r_{c}\left(x\right)=\underset{i}{\text{mean}}\left\{d_{i}^{h_{i}}\left(x\right),i\;f\;f_{i}^{h_{i}}\left(x\right)==c\right\}$ , *c* = *cl*_1, …, *cl*_*n*.

## 4 Application

For assessing the framework presented in Section 3, we conducted a series of experiments using the curated bioactivity NDM-1 database, consisting of 39.75 % non-active, 29.26 % weakly active and 30.99 % strongly active molecules. Firstly, we evaluate the performance of two Multiple Instance Learning classifiers against classical Machine Learning algorithms, subsequently we assess the performance of the ensemble classifier on a held-out test set and demonstrate the generalizability of the framework, and finally, we evaluate the ranking performance of the ensemble classifier. Furthermore, we Virtually Screened the Drugbank [24] for discovering potential strongly active inhibitors among the known human drugs and report the most interesting results.

The numerical representation of the molecules for the classical Machine Learning task was acquired by employing the pre-trained Mol2vec model [39] resulting in 300 dimensional embeddings. 864 unique Morgan identifiers and 21 ‘UKN’ structures were identified in the database. As reported in ref. [39] the vector that corresponds to unknown structures (‘UNK’) tend to be close to the zero vector, and thus it does not contribute in a significant manner (additively) to the resulting molecular representation.

The Multiple Instance Learning representation of compounds, consisting of bags of embedding vectors corresponding to molecular substructures, resulted in 19,082 instances of both radii 0 and 1. Only a small amount of instances (0.29 %) corresponded to unknown structures: 1 and 55 instances of radii 0 and 1 respectively. We removed all instances corresponding to unknown structures, because they refer, likely, to different substructures, and thus their contribution to the molecular representation is limited, if not misleading. After the removal procedure, we verified that there were no empty (i.e. without any instances) molecules in our dataset. Finally, the Multiple Instance Learning representation resulted to 7264 instances of radius 0 and 11,818 instances of radius 1.

For evaluating the ranking and classification performance of the ensemble classifier , we employed a held-out Test Set (TS). This TS was the result of a 90 % (Training Set – TrS) −10 % (TS) stratified split of the database. For the evaluation of the individual classifiers we used the 90 % (TrS) split of the initial database and the models were assessed using a 10-fold Cross Validation procedure.

We chose four state-of-the-art classical Machine Learning algorithms: Support Vector Machines (SVM) with Radial Basis Kernel (RBF), Linear Discriminant Analysis (LDA), Multi-Layer Perceptron (MLP) and Radom Forest (RF) [20] and two Multiple Instance Learning algorithms TensMIL [30] and TensMIL2 [31] for evaluating and compering the classification performance between the classical ML and MIL paradigms.

TensMIL and TensMIL2 are two state-of-the-art Multiple Instance Learning algorithms that consist of two phases: (i) feature extraction from tensors of order greater than 3 and (ii) a classification phase that uses the feature matrix extracted from phase (i). Since the instances’ feature vectors are acquired from the Mol2vec model, as described in Section 3.4.2, we do not possess 3D data, and thus, the feature extraction phase of TensMIL and TensMIL2 is omitted. For the sake of completeness, we will give a short overview of the two algorithms. These algorithms are based on two sequential tasks, one regression procedure in the instances’ space and a classification task in the bags’ space. The first regression task consists of a full quadratic robust regression procedure in the instances’ space (i.e. the substructures’ space), where the training is performed using weak instance labels (instances inherit the bag labels). The output of this phase is the response of the model for each individual instance. Subsequently, the responses are grouped together per bag, and the distributions of the responses per bag, are estimated. The second phase consists of a bag Quadratic Discriminant Analysis classifier that takes as features the estimated responses’ distributions per bag and classifies the bags (i.e. compounds). In contrast to TensMIL, TensMIL2 incorporates in the first phase (i.e. regression in the instances’ space) an instance selection procedure, based on the certainty of the predictions, for discarding non-informative instances. The instance selection criterion is based on the 95 % confidence residual intervals of the true mean response of an instance inside a bag. The interested reader can refer to refs. [30, 31] for a detailed presentation of the algorithms.

In Section 3.5 we stated that the set of homogeneous classifiers that form the ensemble classifier can be obtained by a k-fold Cross Validation procedure, using a hyper-parameter optimization procedure per fold. In this work, we used a Bayes optimization approach for fine-tuning the hyper-parameters like in ref. [30]. Each fold is split, in a stratified manner, in Training (FTr_i_) and Test (FT_i_) sets. Subsequently, a 2-fold Cross Validation procedure is performed on the FTr_i_ Set and the negative 2-fold CV balanced accuracy (Bacc) is optimized by the Bayes optimization method to obtain the best hyper-parameters per fold. Finally, using the learnt hyper-parameters, we assess the evaluation metrics on the FT_i_ sets for each fold. This technique allows us to assess the classification performance of the classifier and in the same time to obtain a set of *k* homogeneous classifiers that can be used as an ensemble classifier that is able to rank the classification outputs per class.

We tuned the following hyper-parameters for the investigated algorithms: for SVM the regularization parameter *C* and the RBF kernel scaling parameter *γ*, for Random Forest the number of Forest Trees, for the MLP the number of the hidden layers, the number of neurons of each layer as well as the activation function (logistic, tanh, relu), for TensMIL, the number of bins used for estimating the bags’ responses distribution *ϑ*_*H*_ and the variance retained of the Principal Component Analysis that was performed on the data matrix in the instances space *ϑ*_*p*_ and finally for TensMIL2 *p* that is equal to *ϑ*_*p*_ and *q* that is a threshold defining the instances selection. TensMIL2 disposes also the non-tunable parameter $\vartheta_{H}^{\text{TensMIL}2}$ that corresponds to *ϑ*_*H*_. This parameter was calculated for each experiment by the mean value of the *ϑ*_*H*_s of TensMIL in each of fold in the corresponding experiment.

The voting approach that we used in this study, for outputting the ensemble classifier’s decisions, was the majority-voting scheme. For each compound, the decisions of the 10 homogeneous classifiers are considered, and the class predicted by the majority of the individual classifiers is associated to the specific compound.

For the performance evaluation of the classifiers and the ensemble classifiers, we used the following metrics: accuracy, balanced accuracy and per class precision, recall and F1-score, measured in a 10-fold Cross Validation procedure. The evaluation of the ranking performance was carried out by the per-class top-k accuracy that is defined as follows: ${\text{TopAcc}}_{c}^{\left(k\right)}=\frac{\#\text{top}-\text{k ranked True Positives}}{k}$ , where *c* is referring to the corresponding class.

### 4.1 Results

In this section, we present the evaluation results of the proposed methods. Firstly, we evaluate the classification efficacy of the Multiple Instance Learning classifiers in comparison to the classical Machine Learning classifiers, as well as the generalization ability of the ensemble classifiers that derive from them. Subsequently, we assess the ranking capability of the ensemble framework. Furthermore, we investigate the classification, generalization and ranking abilities of the Multiple Instance Learning methods, when using only atomic based (i.e. radius 0) molecular representations, as well as when using only substructure representations that involve two atoms (i.e. radius 1 based representations). We compare their classification and speed performance, against the case where all substructures are used for training the models. Finally, we predict the activity and rank, according to the confidence of the ensemble classifier, of drugs in the Drugbank, using the proposed ensemble classification and ranking framework.

#### 4.1.1 Classification evaluation of ML versus MIL models

Table 2, presents the performance evaluation, in terms of average 10-fold Cross Validation metrics, of Machine Learning versus Multiple Instance Learning tasks. Precision, Recall and F-1 score are calculated for each individual class.

**Table 2:** Classification performance evaluation of classical machine learning and Multiple Instance Learning classifiers for NDM-1 activity prediction. Best performances are denoted by bold type.

10-fold cross validation evaluation of classifiers											
	Acc.	Bacc.	Precision			Recall			F1-score		
			Non activity	Weak activity	Strong activity	Non activity	Weak activity	Strong activity	Non activity	Weak activity	Strong activity
SVM	52.25	50.83	59.91	26.78	64.38	63.55	22.55	66.40	59.46	20.21	61.07
LDA	51.09	50.24	65.41	23.01	58.95	57.10	25.63	68.00	57.94	22.43	60.82
RF	52.76	51.15	61.69	23.97	62.41	66.45	24.72	62.28	62.16	21.42	58.61
MLP	50.59	49.41	61.89	29.54	60.18	60.00	23.87	64.35	58.80	22.36	58.41
TensMIL	72.08	70.81	75.14	59.51	80.65	81.29	50.59	**80.53**	77.67	53.57	80.23
TensMIL2	**74.40**	**73.20**	**76.79**	**64.53**	**82.57**	**83.55**	**55.53**	80.52	**79.42**	**58.66**	**81.16**

In terms of Balanced Accuracy, the Multiple Instance Learning algorithms performed from 38.43 %–45.7 % better than the classical Machine Learning algorithms. In the case of strong active compounds, the Multiple Instance Learning approach improved Precision up to 40 % and Recall up to 29.30 %, as compared to the Machine Learning paradigm. The MIL approach had a significant positive effect on the F1-scores in all the classes, compared to the classical ML performance: a 24.95 %–37.07 % improvement for the non-active class, a 138.84 %–190.24 % improvement for the weakly activity class and a 31.36 %–38.47 % for the strong active compounds’ class. We further observed that, in general, the classification performance of all the classifiers in terms of F1-score is poorer for the weakly active class, in comparison to the two other classes. This fact suggests that, as concerning the classification procedure, the boundaries between inactive and weakly active classes, as well as between strongly active and weakly active compounds could not be clearly identified by the classifiers. This fact is not of great concern, for our case, since we primly are interested to discover strongly active NDM-1 inhibitors.

In general, we can observe that the MIL classifiers performed significantly better than the Machine Learning algorithms, in terms of all evaluation metrics. This fact suggests that the Multiple Instance representation, contributed positively to the classification performance. In fact, the initial Mol2vec representation, suitable for classical Machine Learning, does implicitly considers the molecular substructures of each compound, by adding up all vectors corresponding to substructures. In contrast the MIL representation takes in consideration explicitly all the molecular substructures, by forming a bag of substructure vectors. The biding affinity of a ligand to a target, in most of the cases, is not affected by the entire compound, but by specific parts of the molecule (i.e. a subset of its substructures) that have specific molecular structures and properties. In this frame, the MIL representation, involving all molecular substructures and their weak labeling, is beneficial to the classification performance.

Eventually, we contrast the results of ref. [23] to our experiments, even though, the two experimental settings are not fully comparable and we could not fully reproduce the results of ref. [23]. In fact, the authors of ref. [23] use hypothetical inactive compounds (i.e. without experimental validation), the cut-off values, used for the annotation of the known weak and strongly active compounds, are less stricter than the ones used in this study and the molecular representation is done by computing molecular descriptors (i.e. handcrafted features) and not embeddings. Furthermore, from the 511 compounds with known activities that are included in the NDMI database [23], we could identify only 127 molecules that corresponded to publications with a valid DOI number referring to the NDM-1 enzyme. Nonetheless, in terms of the F1-score, TensMIL2 demonstrates 15.36 %–41.66 % improved performance, in comparison to the models in ref. [23], for the strongly activity class. As concern the F1-score performance, for the inactive and weakly active classes, the models in ref. [23] perform better than the proposed models. We can conclude, thus, that the molecular representation, using substructure embeddings in a Multiple Instance Learning frame, as well as the labeling, using stricter cut-off values for the strongly activity class, contributed positively to the classification performance of the strong active class.

#### 4.1.2 Generalization ability of the ensemble classification framework

The generalization ability assessment of the ensemble Multiple Instance Learning classifiers, in comparison to the classical Machine Learning ensemble models, was performed employing an independent test set, different from the datasets used for training, hyper-parameter tuning and Cross Validation assessment of the classifiers. Table 3 resumes the results of this evaluation.

**Table 3:** Classification performance evaluation of the ensemble classifiers on an independent test set for NDM-1 activity prediction. Best performances are denoted by bold type.

10-fold cross validation evaluation of the ensemble classifiers on an independent test set											
	Acc.	Bacc.	Precision			Recall			F1-score		
			Non	Weak	Strong	Non	Weak	Strong	Non	Weak	Strong
			activity	activity	activity	activity	activity	activity	activity	activity	activity
SVM	39.08	32.76	39.29	33.33	0.00	94.29	4.00	0.00	55.46	07.14	Inf.
LDA	35.63	38.00	66.67	42.86	33.77	5.71	12.00	96.30	10.53	18.75	0.5
RF	40.23	33.71	40.00	50.00	0.00	97.14	4.00	0.00	56.67	07.41	Inf.
MLP	48.28	50.67	**100**	50.00	42.19	20.00	32.00	**100**	33.33	39.02	59.34
TensMIL	**75.86**	**73.16**	76.74	**76.92**	74.19	**94.29**	40.00	85.19	**84.62**	**52.63**	**79.31**
TensMIL2	73.56	70.79	75.00	61.11	**80.00**	**94.29**	**44.00**	74.07	83.54	51.16	76.92

In Table 3, we can observe that the classical Machine Learning ensemble classifiers perform poorer, in terms of Accuracy, Balanced Accuracy and F1-score, than the individual classifiers, thus implying that their generalizability capacity is relatively low. In the case of the LDA model we observe that the ensemble classification framework demonstrates a high recall score (96 %) for the strong active class, but a very poor precision score (34 %) for the same class, implying that the False Positive predictions are relatively high. The same applies for the MLP model, that demonstrates a very high recall score for the strong active class (100 %), but a very poor precision score (42.19 %) for the same class. Finally, the ensemble classifiers employing the SVM and Random Forest models were not able to predict any molecules of the strong active class.

In the case of the Multiple Instance Learning ensemble models, we see that the ensemble TensMIL model achieves improved classification results, in terms of Accuracy, Balanced Accuracy and Recall for the strongly active class. In terms of the F1-score the ensemble TensMIL model performed better for the non-activity and weak activity classes and slightly worse (less than 1 %), for the strong activity class, when compared to the individual models. The ensemble TensMIL2 model performed slightly worse, (but comparable), compared to the initial model. This fact implies that the initial Multiple Instance Learning models have good generalization capabilities through the ensemble classification scheme. In general, the Multiple Instance Learning ensemble models achieved 86.29 %–123.32 % better classification performance, in terms of Balanced Accuracy, when compared to the classical Machine Learning ensemble models.

**Figure 5:**
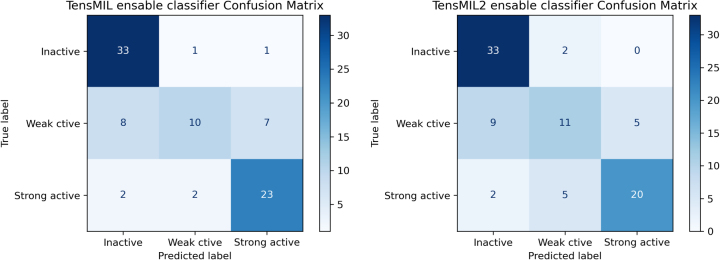
Confusion matrices of TensMIL and TensMIL2, for the ensemble classifier on the independent test set.

In general, we can state that the Multiple Instance Learning classifiers improved the classification results, when compared to the Machine Learning models, both in the individual, as well as in the ensemble models. Finally, in terms of the F1-score of the Multiple Instance Learning ensemble framework , we observed that the performance for the inactive and strongly active classes was significantly better, than for the weakly active class. The same behavior was also observed for the individual classifiers and suggests that the task of distinguishing between the weak active versus the inactive and strongly active classes is a hard classification task. The confusion matrices for the ensemble Multiple Instance Learning frameworks are presented in Figure 5.

#### 4.1.3 Ranking performance assessment of the ensemble classification frameworks

Table 4 resumes the evaluation outcomes in terms of top-3, 5, 10 and 15 ranking accuracy of the ensemble classification frameworks.

**Table 4:** Evaluation of the per class ranking performance in terms of top-k accuracy of the ensemble classifiers, for ranking the predictions of NDM-1 activity. Best performances are denoted by bold type.

	Inactive class				Weak active class				Strong active class			
	Top-3	Top-5	Top-10	Top-15	Top-3	Top-5	Top-10	Top-15	Top-3	Top-5	Top-10	Top-15
SVM	33.33	60	60	60	33.33	20	10	6.67	0	0	0	0
LDA	66.67	40	20	13.33	**66.67**	60	30	20	33.3	60	70	60
RF	33.33	40	50	46.67	33.33	20	10	6.67	0	0	0	0
MLP	**100**	**100**	70	46.67	**66.67**	40	70	53.33	**100**	**100**	**100**	**93.33**
TensMIL	**100**	80	**90**	**86.67**	**66.67**	60	**80**	**66.67**	**100**	**100**	90	**93.33**
TensMIL2	**100**	80	**90**	**93.33**	**66.67**	**80**	60	60	**100**	**100**	90	**93.33**

As measured by top-k accuracy, the Multiple Instance Learning frameworks demonstrate an improvement from 1.33 to 7 times, for the non-active class and up to 10 and 3 times for the weakly and strongly active classes respectively, when compared to the classical Machine Leaning paradigm. Furthermore, for the two Multiple Instance Learning frameworks, the top-5 ranked strong active compounds correspond to real strong active compounds (i.e. the top-3 and top-5 accuracies are 100 %). The MLP model, displays similar behavior in terms of top-3 to top-5 accuracies for the Inactive and Strong active class, and a slightly worser behavior for the Weak active class, in comparison to the MIL models. Furthermore, as expected, Random Forest and SVM frameworks did not ranked at the top-15 any strong active compound. Finally, we observed, as in the classification task, that in general, the Multiple Instance Learning frameworks demonstrated improved raking performance in comparison to the Machine Learning frameworks, and again the ranking evaluation yielded improved results for the non-active and strongly active classes as for the weakly active class.

#### 4.1.4 Using molecular representations based on radius 0 or radius 1 substructures

The molecular representation, in the frame of Multiple Instance Learning, using substructure embeddings corresponding to all available substructures (i.e. substructures of radii zero and one) is beneficial for the classification and ranking performance, but it results to a big amount of instances, slowing thus the training and inference procedures (see Table 8). In this section, we investigate the influence on the classification and ranking performance, when using Multiple Instance Learning representations, using only atomic (i.e. radius 0) or only bi-atomic (i.e. radius 1) molecular representations. In fact, in our case, radius 0 representations produce 2.63 times less instances and radius 1 representations produce 1.61 times less instances with respect to the full representation using both radii.

As presented in Table 5, TensMIL classifier, for radius 0 representations, demonstrates a reduction of 10.11 %, 12.35 % and 10.52 % in terms of Accuracy, Balanced Accuracy and F1-score for the strong active class respectively. The same effect is observed for TensMIL2, where the reduction in the performance is of 15.16 %, 16.76 % and 13.73 % in terms of the same evaluation metrics. In contrast, the representation of the molecules, using only embeddings of radius 1 substructures, seems to have not any significant effect, on all the classification evaluation metrics, for both TensMIL and TensMIL2.

**Table 5:** Classification performance evaluation of classical versus multiple instance learning classifiers, using only substructures of radius zero or one for NDM-1 activity prediction. Best performances are denoted by bold type.

10-fold cross validation evaluation of classifiers											
	Acc.	Bacc.	Precision			Recall			F1-score		
			Non activity	Weak activity	Strong activity	Non activity	Weak activity	Strong activity	Non activity	Weak activity	Strong activity
TensMIL radius = 0	64.79	62.06	61.97	66.83	73.55	**86.45**	27.51	72.23	71.61	35.66	71.79
TensMIL2 radius = 0	63.12	60.93	62.41	**67.83**	67.41	79.68	27.11	76.00	68.84	36.66	70.02
TensMIL radius = 1	72.95	71.62	74.90	62.52	**81.19**	82.90	52.19	79.77	78.45	56.37	80.06
TensMIL2 radius = 1	**73.97**	**72.79**	**76.68**	64.11	79.44	82.58	**53.97**	**81.82**	**79.29**	**58.32**	**80.16**

As demonstrated in Table 6, when comparing the classification efficacy of the ensemble classifiers on an independent test set, the radius zero representations show a diminishing efficacy by 19.69 %, 22.76 % and 20.88 % in terms of Accuracy, Balanced Accuracy and F1-score for the strong activity class respectively. In contrary radius 1 representations display similar classification capacity behavior as the representation using both radii.

**Table 6:** Classification performance evaluation of the ensemble classifiers on an independent test set using only substructures of radius zero ore one for NDM-1 activity prediction. Best performances are denoted by bold type.

10-fold cross validation evaluation of the ensemble classifiers on an independent test set											
	Acc.	Bacc.	Precision			Recall			F1-score		
			Non activity	Weak activity	Strong activity	Non activity	Weak activity	Strong activity	Non activity	Weak activity	Strong activity
TensMIL radius = 0	60.92	56.51	58.93	57.14	66.67	94.29	16.00	59.26	72.53	25.00	62.75
TensMIL2 radius = 0	59.77	55.46	57.14	60.00	65.38	91.43	12.00	62.96	70.33	20.00	64.15
TensMIL radius = 1	74.71	72.30	**82.05**	61.11	73.33	**91.43**	**44.00**	81.48	**86.49**	51.16	77.19
TensMIL2 radius = 1	**75.86**	**73.54**	80.00	**64.71**	**76.67**	**91.43**	**44.00**	**85.19**	85.33	**52.38**	**80.70**

Similar behavior can be observed with TensMIL classifier, with the difference that the radius 1 representation displays an increase of 15 % in the Recall of the strong active class, in respect to the full representation of the molecules.

In Table 7, the ranking results of the ensemble frameworks, on an independent test set are presented. Here we can observe that the ranking efficacy of the TensMIL ensemble framework, displays 100 % top-3 to top-10 accuracy, for the strong activity class, in the case of radius 0 representations, and 100 % top-3 to top-15 accuracy in the case of radius 1 representations. In the case of TensMIL2 the absolute score, for the strong active class, is obtained for the top-3 to top-5 accuracies in the case of radius 0, and for the top-3 to top-10 accuracies in the case of radius 1 representations.

**Table 7:** Evaluation of the per class ranking performance in terms of top-k accuracy of the ensemble classifiers, using substructures of radius zero or radius one, for ranking the predictions of NDM-1 activity. Best performances are denoted by bold type.

	Inactive class				Weak active class				Strong active class			
	Top-3	Top-5	Top-10	Top-15	Top-3	Top-5	Top-10	Top-15	Top-3	Top-5	Top-10	Top-15
TensMIL radius = 0	**100**	**100**	**100**	73.33	**66.67**	**60**	40	26.67	**100**	**100**	**100**	86.67
TensMIL2 radius = 0	**100**	**100**	80	73.33	**66.67**	**60**	30	20	**100**	**100**	80	80
TensMIL radius = 1	66.67	60	80	86.67	**66.67**	**60**	60	60	**100**	**100**	**100**	**100**
TensMIL2 radius = 1	**100**	**100**	**100**	**93.33**	**66.67**	**60**	**70**	**66.67**	**100**	**100**	**100**	93.33

Table 8, resumes the mean training (over the 10 train folds) and the mean testing (over the 10 test folds) of TensMIL and TensMIL2, for the full (i.e. radius 0 and radius 1), the radius 0 and the radius 1 representations. It can be easily observed that the training and testing time for the radius 0, in relation to the full molecular representations, is 98.01 and 7.99 times faster. In the case of radius 1 representations, we have 2.98 times faster training time and 37.40 % faster testing time, relatively to the full representation. Therefore, it is obvious that the radius 0 representations produce models, which achieve about 10 % purer classification performance. In the case of radius 1 representations, the gain in training and testing time is about 3 and 2 times in comparison to the full representation, without significant loss of the classification efficacy of the individual and the ensemble classifiers. Finally, radius 1 representation seems to benefit the ranking performance of both TensMIL and TensMIL2 ensemble models.

**Table 8:** Mean training time across the 10-folds and mean testing time in seconds, for the training and testing of the multiple instance learning algorithms employing (i) all the available substructures (radii 0 and 1) (ii) only atomic substructures (radius 0) and (iii) only atomic substructures with their first neighbors (radius 1). Best performances are denoted by bold type.

Mean training and testing times in seconds						
	Radii 0 and 1		Radius 0		Radius 1	
	Train (sec.)	Test (sec.)	Train (sec.)	Test (sec.)	Train (sec.)	Test (sec.)
TensMIL	55.8242	0.26837	**0.56956**	**0.021869**	18.7401	0.14098
TensMIL2	9.4581	0.052014	**1.1836**	**0.043989**	18.9675	0.13906

#### 4.1.5 Virtual screening for NMD-1 inhibitors in the Drugbank

We Virtually Screened 11,290 drugs of the Drugbank, for NDM-1 inhibitors, employing the Multiple Instance Learning ensemble classifier that consists of 10 TensMIL individual classifiers. We used both radius 0 and radius 1 substructures embeddings, employing the pre-trained Mol2vec model of ref. [39]. Out of 11,290 compounds of the database 197 had only unknown structures, thus we could not make any prediction for them. From the remaining 11,093, 9433 (85.04 %) were classified as inactive, 1115 (10.05 %) as weakly active and 545 (4.91 %) as strongly active. Table 9 resumes the 15 top-ranked compounds, by order of significance, of the strong active class, as predicted by the proposed framework. In the top-15, ranked as strongly active drugs, we identified 9 experimental, 4 approved, 1 investigational and 1 illicit drug.

**Table 9:** The 15 top-ranked strongly active compounds from the Drugbank, ranked by order of significance, as predicted by the proposed ranking and classification framework employing the TensMIL algorithm.

Ranked strongly active compounds as predicted by the TensMIL ensemble ranking and classification framework			
DB id	Generic name	Canonical SMILES	Ranking score
DB13659	Tenonitrozole	O=C(Nc1ncc([N+](=O)[O-])s1)c1cccs1	0.999905777
DB09175	Mirfentanil	O=C(c1ccco1)N(c1cnccn1)C1CCN(CCc2ccccc2)CC1	0.99988165
DB03099	5-Amino 6-Nitro Uracil	Nc1c([N+](=O)[O-])[nH]c(=O)[nH]c1=O	0.999855499
DB14719	Bentazepam	O=C1CN=C(c2ccccc2)c2c(sc3c2CCCC3)N1	0.999791661
DB14028	Nordazepam	O=C1CN=C(c2ccccc2)c2cc(Cl)ccc2N1	0.999706118
DB01511	Delorazepam	O=C1CN=C(c2ccccc2Cl)c2cc(Cl)ccc2N1	0.999704894
DB06075	Linsitinib	C[C@]1(O)C[C@@H](c2nc(-c3ccc4ccc(c5ccccc5)nc4c3)c3c(N)nccn32)C1	0.999687611
DB06228	Rivaroxaban	O=C(NC[C@H]1CN(c2ccc(N3CCOCC3=O)cc2)C(=O)O1)c1ccc(Cl)s1	0.999685463
DB00897	Triazolam	Cc1nnc2n1-c1ccc(Cl)cc1C(c1ccccc1Cl)=NC2	0.999551343
DB00404	Alprazolam	Cc1nnc2n1-c1ccc(Cl)cc1C(c1ccccc1)=NC2	0.999528359
DB09180	Thienylfentanyl	CCC(=O)N(c1ccccc1)C1CCN(CCc2cccs2)CC1	0.999416387
DB15495	Rocaglamide	COc1ccc([C@@]23Oc4cc(OC)cc(OC)c4[C@]2(O)[C@H](O)[C@H](C(=O)N(C)C)[C@H]3c2ccccc2)cc1	0.999326891
DB14717	Nitrazolam	Cc1nnc2n1-c1ccc([N+](=O)[O])cc1C(c1ccccc1)=NC2	0.999131403
DB14174	Dipentamethylenethiuram disulfide	S=C(SSC(=S)N1CCCCC1)N1CCCCC1	0.998987551
DB14716	Clonazolam	Cc1nnc2n1-c1ccc([N+](=O)	0.998949098
		[O-])cc1C(c1ccccc1Cl)=NC2	

## 5 Discussion

The creation of a novel database of compounds with experimental activity properties against NDM-1, along with the established labelling procedure that can handle multiple activity properties, curate inconsistencies due to different experimental settings and label the molecules in three classes using stricter cut-off values has been proven beneficial for the discovery of new NDM-1 inhibitors. Moreover, the use of the Multiple Instance Learning representation of molecules, using substructure embeddings, had a positive effect on the 3-class activity classification performance, in comparison to the classical molecular representation, where each compound is represented by only one embedding vector. This can be attributed to the fact that the binding affinity of a compound implicates a part of the molecule (i.e. a subset of its substructures) and as the substructures are explicitly represented in the bag representation of a compound, this acts beneficial to the classification performance.

The ensemble ranking and classification framework, based on the Multiple Instance Learning models (TensMIL and TensMIL2) displayed promising generalization abilities, in comparison to the ensemble models based on classical Machine Learning algorithms. Furthermore, our experiments employing bag representations consisting only of radius 0 or radius 1 substructures, revealed that, the classification efficiency of the classifiers as well as of the ensemble classifiers using radius 1 representations, were not affected, in comparison to the case where the bags are represented using both radii (i.e. 0 and 1), but the training and testing time of the models were significantly better. The ranking accuracy of TensMIL ensemble classifier, in terms of top-3 to top-15 accuracy, when using only radius 1 substructures embeddings for representing compounds, for the strong active class was 100 %, in an independent test set, suggesting that the top-15 ranked compounds were indeed strongly active. Finally, we scanned the Drugbank, a database comprising known human drugs, for strong active compounds and we delivered the top-15 ranked strongly active compounds.

In future work, other molecular representations (e.g. molecular representations based on molecular graphs, or representations comprising possible multimodal, [i.e. structural and physicochemical], information) could be explored and tensor decompositions or other multimodal data fusion methods could be exploited, for extracting discriminating features for the activity classification task, either in the frame Multiple Instance or classical learning. Finally, an interesting aspect that requires further investigation is the study of substructure contributions on the activity prediction task, using local interpretability methods on the MIL setting. Model agnostic interpretability methods [49], or interpretability methods directly adapted to TensMIL or TensMIL2, could reveal interesting contributions of the molecular substructures to the activity classification task.
